# Effectiveness of immune checkpoint inhibitor therapy on bone metastases in non-small-cell lung cancer

**DOI:** 10.3389/fimmu.2024.1379056

**Published:** 2024-06-18

**Authors:** Annalise G. Abbott, Daniel E. Meyers, Golpira Elmi-Assadzadeh, Igor Stukalin, Alessandro Marro, Shannon K. T. Puloski, Don G. Morris, Winson Y. Cheung, Michael J. Monument

**Affiliations:** ^1^ Section of Orthopaedic Surgery, University of Calgary, Calgary, AB, Canada; ^2^ Arnie Charbonneau Cancer Institute, University of Calgary, Calgary, AB, Canada; ^3^ Department of Oncology, University of Calgary, Calgary, AB, Canada; ^4^ McCaig Bone & Joint Institute, University of Calgary, Calgary, AB, Canada; ^5^ Departmenmt of Radiology, University of Calgary, Calgary, AB, Canada

**Keywords:** non-small-cell lung cancer, bone, metastasis, checkpoint inhibitors, tumour microenvironment

## Abstract

**Background:**

Bone metastases (BoMs) are prevalent in patients with metastatic non-small-cell lung cancer (NSCLC) however, there are limited data detailing how BoMs respond to immune checkpoint inhibitors (ICIs). The purpose of this study was to compare the imaging response to ICIs of BoMs against visceral metastases and to evaluate the effect of BoMs on survival.

**Materials and methods:**

A retrospective, multicentre cohort study was conducted in patients with NSCLC treated with nivolumab or pembrolizumab in Alberta, Canada from 2015 to 2020. The primary endpoint was the real-world organ specific progression free survival (osPFS) of bone versus visceral metastases. Visceral metastases were categorized as adrenal, brain, liver, lung, lymph node, or other intra-abdominal lesions. The secondary outcome was overall survival (OS) amongst patients with and without BoMs.

**Results:**

A total of 573 patients were included of which all patients had visceral metastases and 243 patients (42.4%) had BoMs. High PD-L1 expression was identified in 268 patients (46.8%). No significant difference in osPFS was observed between bone, liver, and intra-abdominal metastases (p=0.20 and p=0.76, respectively), with all showing shorter osPFS than other disease sites. There was no difference in the osPFS of extra-thoracic sites of disease in patients with high PD-L1 expression. There was significant discordance between visceral disease response and bone disease response to ICI (p=0.047). The presence of BoMs was an independent poor prognostic factor for OS (HR 1.26, 95%CI: 1.05–1.53, p=0.01).

**Conclusion:**

Metastatic bone, liver, and intra-abdominal lesions demonstrated inferior clinical responses to ICI relative to other sites of disease. Additionally, the presence of bone and liver metastases were independent poor prognostic factors for overall survival. This real-world data suggests that BoMs respond poorly to ICI and may require treatment adjuncts for disease control.

## Introduction

Immune checkpoint inhibitors (ICIs) are revolutionizing the treatment landscape of several solid tumour malignancies, including non-small-cell lung cancer (NSCLC). ICIs targeting the programmed death-1 (PD-1)/PD-ligand 1 (PD-L1) axis are now standard of care for metastatic NSCLC in the first- and second- line setting (1–9).

Bone metastases (BoMs) are highly prevalent in NSCLC, with up to 40% of patients developing BoMs during the course of their disease (10). BoMs will frequently cause skeletal-related events (SREs) such as intractable bone pain, neurologic compromise, hypercalcemia, and pathologic fracture leading to reductions in Eastern Cooperative Oncology Group Performance Status (ECOG PS) and quality of life (11–13). Furthermore, the presence of BoMs is a poor prognostic factor for overall survival (OS) (14). Insights into whether BoMs respond to systemic therapies such as ICIs are important to multidisciplinary decision making, and may prevent unnecessary intervention. Conversely, lesions not anticipated to heal can be strategically treated with radiotherapy (RT) or orthopaedic surgery to prevent progressive morbidity.

There is growing evidence that the anatomic site of metastatic disease affects response to ICI (15, 16). Pre-clinical and clinical studies have demonstrated distinct patterns of organ-specific responsiveness (17–28). This may be due to differences in the tumour biology of malignant cells that metastasize to different organs and variations in the tumour-immune microenvironment (TIME) intrinsic to the cell populations of different anatomic tissues (29–32). The alteration in normal bone homeostasis with BoMs creates physical space for tumour expansion and induces the release of growth factors and cytokines that further support tumour growth and an immunosuppressive TIME (13, 33). Mechanisms of immunosuppression within the bone TIME include a decreased population of cytotoxic T cells and natural killer (NK) cells, increased populations of suppressor cells including regulatory T cells (Tregs) and myeloid-derived suppressor cells (MDSC), and a cytokine environment favouring tumour growth (13, 22, 34, 35). This is largely driven by supraphysiologic levels of tissue growth factor beta (TGF-β) released from bone resorption (22).

Preliminary studies have identified inferior clinical outcomes and lower therapeutic response rates in patients with BoMs treated with ICIs, suggesting ICIs may be less effective in BoMs (15, 24, 36). In a recent study of 1959 patients with advanced NSCLC treated with nivolumab, there was shorter progression free survival (PFS), lower OS and a lower objective response rates (ORR) in the subgroup of patients with BoMs (24). Poor clinical outcomes in this population have been similarly observed in other smaller retrospective studies (36, 37). Few of the large randomized controlled trials have specifically investigated the consequences of BoMs on ICI response, nor stratified patients according to the presence of BoMs. Thus, evidence specifically addressing patients with BoMs is lacking, and the prognostic significance of BoMs with ICI treatment remains unclear.

In this study, the imaging-based treatment responses of individual sites of metastatic disease were systematically evaluated in patients with NSCLC treated with ICI to determine site-specific patterns of response. The principal outcome measure compared the real-world organ specific PFS (osPFS) of metastatic bone lesions against visceral and lymphatic lesions. Secondary outcomes evaluated OS of patients with and without BoMs.

## Materials and methods

The records of all patients who received ICI therapy advanced NSCLC in Alberta, Canada between December 2015 and June 2020 were retrospectively reviewed. The last follow-up evaluation of patients was performed in July 2021. Patients were identified for potential inclusion from the Alberta Immunotherapy Database developed utilizing the Alberta Cancer Registry (38). Adult patients who received at least one dose of ICI for metastatic NSCLC were screened for inclusion. Patients were excluded if they had insufficient imaging, locally advanced NSCLC or a second, active malignancy. Adequate imaging was defined as: (1) a baseline total body CT scan of the chest, abdomen and pelvis (CT CAP) or positron emission tomography (PET) scan within three months prior to starting treatment and, (2) regular surveillance imaging at a minimum of every three months while on treatment. Consequently, only BoMs of the spine, ribs, pelvis, and proximal femur were routinely evaluated. Patients received ICI according to local practices in a real-world setting and additionally, they may have received additional treatments such as RT, surgery and/or other medications according to clinical indications. This study was conducted in accordance with the Declaration of Helsinki (1965) and approved by the local Research Ethics Board (HREBA.CC-19–0380). A waiver of consent was obtained for this retrospective study.

Tumour response to ICI treatment was assessed across serial imaging studies. Visceral tumour response was evaluated by retrospectively applying parameters according to the Response Evaluation Criteria in Solid Tumors (RECIST) version 1.1 (**Table 1**) (39). The best response of each metastatic organ of disease was characterized individually according to the sum of all lesions within that organ. Visceral organ sites of disease were categorized by anatomic site as adrenal, brain, liver, lung, lymph node or other intra-abdominal lesions. As bone lesions are generally considered immeasurable by RECIST, they were analysed separately following the subjective measures of the MD Anderson (MDA) criteria (**Table 1**) (40). An MSK radiologist independently reviewed the bone response of any imaging study that was deemed indeterminate by the previous radiology report. Metastatic lesions in irradiated areas either before or during ICI treatment were not evaluated for response unless there had been documented disease progression with ICI at that site to reduce the confounding effect of radiation. Time to progression of each organ site of disease was evaluated to determine the real-world organ specific (os) PFS. Best ORR, PFS and OS were evaluated. Complications including immune-related adverse events (IrAE) and SREs including surgery, RT, and spinal cord compression were recorded. Sub-group analysis was performed in patients with high PD-L1 expression. PD-L1 is routinely analysed on pathology samples at a single laboratory for the entire health region utilizing the PD-L1 IHC 22C3 pharmDx assay by Dako (Agilent Technologies, California).

**Table 1:** Response criteria for visceral and bone lesions.

Response	Imaging Criteria
*Visceral Tumours* [10]	
CR	Disappearance of all lesions Reduction to <10mm in short axis for all lymph node metastases
PR	>30% reduction in diameter
PD	≥20% growth in diameter Appearance of new lesions
SD	Neither CR, PR nor PD
*Bone Tumours* [13]	
CR	Complete sclerotic fill in Normalization of bone density
PR	Development of a sclerotic rim, partial sclerotic fill-in, osteoblastic flare ≥50% subjective decrease in size
PD	New bone metastases ≥25% subjective increase in size
SD	No change

CR, complete response; PR, partial response; PD, progressive disease; SD, stable disease.

### Statistical analysis

Descriptive statistics were performed for all patient characteristics. OS and PFS were calculated using the Kaplan-Meier survival analysis with 95% confidence intervals (CI). OS and PFS were measured from the first dose of ICI. PFS was calculated as the time from the start of ICI treatment until evidence of progressive disease or death, whichever occurred first. Patients who were lost to follow-up or alive at the time of data collection were censored at the last date of follow-up. Differences between survival curves were evaluated with log rank tests. Real-world osPFS was estimated using Kaplan-Meier survival for each site of metastatic disease and compared using pairwise log-rank tests. Discordance between bone and visceral response were compared with a McNemar test. Categorical variables were compared using Chi-square tests. A Cox proportional hazards model was used to evaluate the association between patient characteristics and survival; Hazard Ratios (HRs) with 95% CIs were reported. A subgroup analysis was performed for patients with PD-L1 expression ≥50%. All statistical analyses were performed using Lifelines and Scipy libraries in Python.

## Results

A total of 743 patients with metastatic NSCLC were treated with ICI across five centres in Alberta, Canada over a five-year period. Among them, 573 patients had sufficient imaging and were included in this study. Median age was 66.9 years and 295 patients (51.5%) were male. Adenocarcinoma was the most common histologic subtype (n=448, 78.2%). On baseline imaging, 243 patients (42.4%) had evidence of BoMs. 522 patients (91.1%) received ICI monotherapy, with 191 patients receiving nivolumab and 308 receiving pembrolizumab for a median of five cycles (IQR: 2–16). Combined pembrolizumab and platinum doublet therapy was prescribed in 51 patients (8.9%). Most patients received an ICI as first-line (n=285, 49.7%) or second-line treatment (n=228, 39.8%). Driver mutations, including EGFR, KRAS, and ALK were present in 49 patients (n=24, 23, and 2 patients respectively). All patients with an EGFR driver mutation were treated in the second or subsequent line setting after receiving a tyrosine kinase inhibitor as first line. The median frequency of follow-up imaging studies was 7.6 weeks (IQR: 6.4–9.9). Baseline patient and treatment characteristics are summarized in **Table 2**.

**Table 2:** Patient and treatment characteristics.

	Total Cohort n=573	BoMs Group n=243	No BoMs Group n=330	p-value
Median age (IQR)	66.9 (60.6 – 73.4)	66.0 (59.5–72.2)	68.9 (62.0–74.0)	0.01^*^
Sex, n (%)				0.13
Male	295 (51.5)	134 (55.1)	161 (48.8)	
Female	278 (48.5)	109 (44.9)	169 (51.2)	
Histology, n (%)				0.04^*^
Adenocarcinoma	448 (78.2)	202 (83.1)	246 (74.6)	
Squamous cell carcinoma	104 (18.2)	37 (15.2)	67 (20.3)	
Unknown	21 (3.7)	4 (1.7)	17 (5.2)	
ECOG PS, n (%)				0.69
0	70 (12.3)	25 (10.4)	45 (13.7)	
1	333 (58.6)	142 (59.2)	191 (58.1)	
2	140 (24.7)	62 (25.8)	78 (23.7)	
3	25 (4.6)	11 (4.6)	15 (4.6)	
Sites of metastatic disease at baseline, n (%)				
Lung	552 (96.3)	231 (95.1)	321 (97.3)	0.16
Lymphatic	427 (74.5)	179 (73.7)	248 (75.2)	0.68
Bone	243 (42.4)	243 (100)	0 (0)	
Brain	104 (18.2)	49 (20.2)	55 (16.7)	0.29
Liver	122 (21.3)	75 (61.5)	168 (27.3)	<0.001^*^
Adrenal	106 (18.5)	48 (19.8)	58 (17.6)	0.51
Other intra-abdominal	49 (8.6)	24 (9.9)	25 (7.6)	0.33
PD-L1 Status				0.051
None	80 (14.0)	38 (15.6)	42 (12.7)	
Low (1–49%)	95 (16.6)	42 (17.3)	53 (16.1)	
High (≥50%)	268 (46.8)	98 (40.3)	170 (51.5)	
Unknown	130 (22.7)	65 (26.8)	65 (19.7)	
Driver mutation, n(%)				
EGFR	24 (4.2)	16 (6.7)	6 (1.8)	0.006^*^
KRAS	23 (4.0)	7 (14.0)	16 (20.0)	0.38
ALK	2 (0.4)	0	2	
Line of treatment, n (%)				0.004^*^
First	285 (49.7)	108 (44.4)	177 (53.6)	
Second	228 (39.8)	99 (40.7)	129 (39.1)	
Subsequent	60 (10.5)	36 (14.8)	24 (7.2)	
Number of cycles, median (IQR)	5 (2–16)	4 (2–10)	7 (2–20)	<0.001^*^
Treatment regimen, n (%)				
Nivolumab	191 (33.3)	89 (36.6)	102 (30.9)	0.15
Pembrolizumab	308 (53.8)	142 (58.4)	217 (65.8)	0.07
Durvalumab	3 (0.5)	0	3 (100)	
Atezolizumab	8 (1.4)	7 (2.9)	1 (0.3)	0.009^*^
Pembrolizumab + platinum doublet	51 (8.9)	25 (10.3)	26 (7.9)	0.32
Concurrent radiation therapy to any site, n (%)^a^	146 (26.9)	95 (41.5)	51 (16.3)	<0.001^*^
Concurrent BMA, n(%)				
Bisphosphonate		11 (4.5)		
Denosumab		7 (2.9)		

BoMs, bone metastases; IQR, interquartile range; ECOG PS, Eastern Cooperative Oncology Group Performance Status; PD-L1, programmed death-ligand 1; EGFR, epidermal growth factor receptor; KRAS, Kirsten rat sarcoma viral oncogene; ALK, anaplastic lymphoma kinase; BMA, bone modifying agent.

a Metastatic lesions in irradiated areas either before or during ICI treatment were not evaluated for response unless there had been documented disease progression with ICI at that site.

### Treatment outcomes

In all patients, the ORR was 31.4% (95%CI: 27.2–36.0). Median PFS of all patients was 3.7 months (95%CI: 3.1–4.3) and OS was 8.1 months (95%CI: 6.9–9.3).

### Organ specific progression free survival

**Figure 1:**
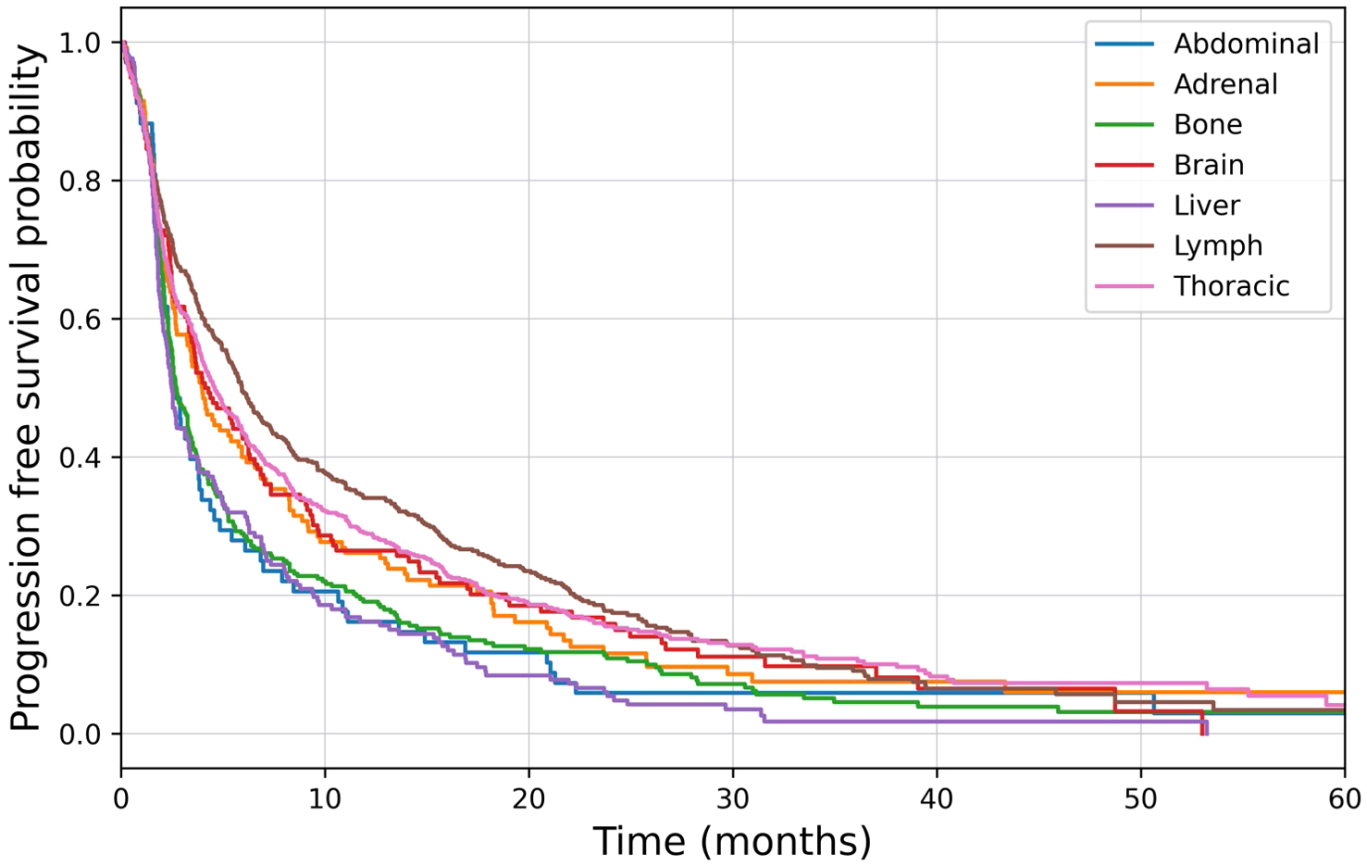
Kaplan-Meier survival analysis of organ specific progression free survival.

Median time to bone lesion progression was 2.7 months (95%CI: 2.4–3.3), significantly shorter than visceral lesion progression at 3.8 months (95%CI: 3.1–4.4 [p=0.01]). To evaluate organ-specific responsiveness to ICI, real-world osPFS amongst each major site of disease was compared using Kaplan-Meier survival analysis (**Figure 1**). A significant difference was found in the time to progression between organs (p<0.005). The shortest one-year PFS was noted in liver (15.7%, 95%CI: 10.7–21.5), abdominal (16.2%, 95%CI: 8.6–25.8) and bone (17.8%, 95%CI: 13.6–22.4) lesions. Pairwise log rank tests demonstrated no significant difference between these sites of disease (p=0.20 and p=0.75, respectively), and all demonstrated shorter osPFS than the other sites of disease. Brain, adrenal, lung lesions, and lymph nodes had a significantly longer PFS than bone lesions (**Table 3**). Furthermore, there was statistically significant discordance between bone disease response and visceral disease response (**Table 4**, p=0.047).

**Table 3:** One-year progression free survival probability by organ site of disease.

Organ site	One-year PFS (%)	Pairwise log-rank test comparison to BoMs, p-value
Abdominal	16.2% (8.6–25.8)	0.40
Adrenal	26.2% (19.0–33.9)	0.03^*^
Bone	17.8% (13.6–22.4)	
Brain	26.3% (19.2–33.9)	0.03^*^
Liver	15.7% (10.7–21.5)	0.20
Lung	30.2% (26.4–34.1)	<0.005^*^
Lymph	34.3% (30.0–38.7)	<0.005^*^

^*^Indicates statistical significance, p<0.05.

PFS, progression free survival; BoMs, bone metastases.

**Table 4:** Response of visceral disease and bone disease to immune checkpoint inhibitors.

	Visceral Disease	Bone Disease
Progressive Disease, n (%)	167 (29.1)	140 (57.6)
Stable Disease, n (%)	152 (26.5)	55 (22.6)
Partial Response, n (%)	145 (25.3)	47 (19.3)
Complete Response, n (%)	0	0

Visceral disease measured retrospectively with RECIST criteria; bone disease measured with MD Anderson criteria.

Significant discordance between visceral disease response and bone disease response was identified, p=0.047.

### Bone metastases and survival

Patients with BoMs had shorter survival compared to those without with a median OS of 6.1 months (95%CI: 4.9–7.1) versus 10.8 months (95%CI: 8.4–14.6; p=0.01) on univariate analysis. Multivariate log rank test demonstrated a difference in OS according to PD-L1 status amongst patients with BoMs (p<0.005). Patients with low PD-L1 expression had significantly shorter survival compared to no PD-L1 and high PD-L1 expression (3.3 months versus 6.3 and 6.9 months respectively, p=0.03 and p<0.005). The presence of BoMs was an independent, poor prognostic factor of OS on multivariate analysis (hazard ratio: 1.26, 95%CI: 1.05–1.53; p=0.01). ECOG PS ≥2, line of treatment ≥2 and the presence of brain, liver and intra-abdominal lesions were also found to be poor prognostic factors for OS (**Table 5**).

**Table 5:** Multivariate analysis of overall survival in entire cohort and high PD-L1 expression subgroup.

Variables	Entire cohort, HR (95%CI); p-value	High PD-L1 expression subgroup, HR (95%CI); p-value
Age	0.99 (0.98–1.00), p=0.24	1.00 (0.99–1.02), p=0.87
Sex, male vs. female	1.01 (0.85–1.22), p=0.87	0.95 (0.72–1.27), p=0.75
Line of treatment, second and subsequent lines vs. first line	1.39 (1.67–3.47), p<0.005^*^	0.85 (0.59–1.22), p=0.38
ECOG PS, ≥2 vs. 0–1	1.97 (1.62–2.40), p<0.005^*^	2.12 (1.58 – 2.85), p <0.005^*^
Baseline lesions, yes vs. no		
Abdominal	1.41 (1.03–1.93), p=0.03^*^	1.43 (0.86–2.38), p=0.17
Adrenal	1.04 (0.82–1.30), p=0.77	1.06 (0.74–1.51), p=0.75
Brain	1.26 (1.00–1.60), p=0.05	1.30 (0.90–1.88), p=0.17
Bone	1.30 (1.08–1.57), p=0.01^*^	1.24 (0.92–1.68), p=0.16
Liver	1.42 (1.14–1.77), p<0.005^*^	1.68 (1.17–2.42), p<0.005^*^
Lung	1.43 (0.86–2.36), p=0.16	1.67 (0.81–3.42), p=0.16
Lymph node	1.19 (0.97–1.46), p=0.10	1.21 (0.84–1.74), p=0.31

^*^Indicates statistical significance, p<0.05.

ECOG PS, Eastern.

### High PD-L1 expression subgroup analysis

**Figure 2:**
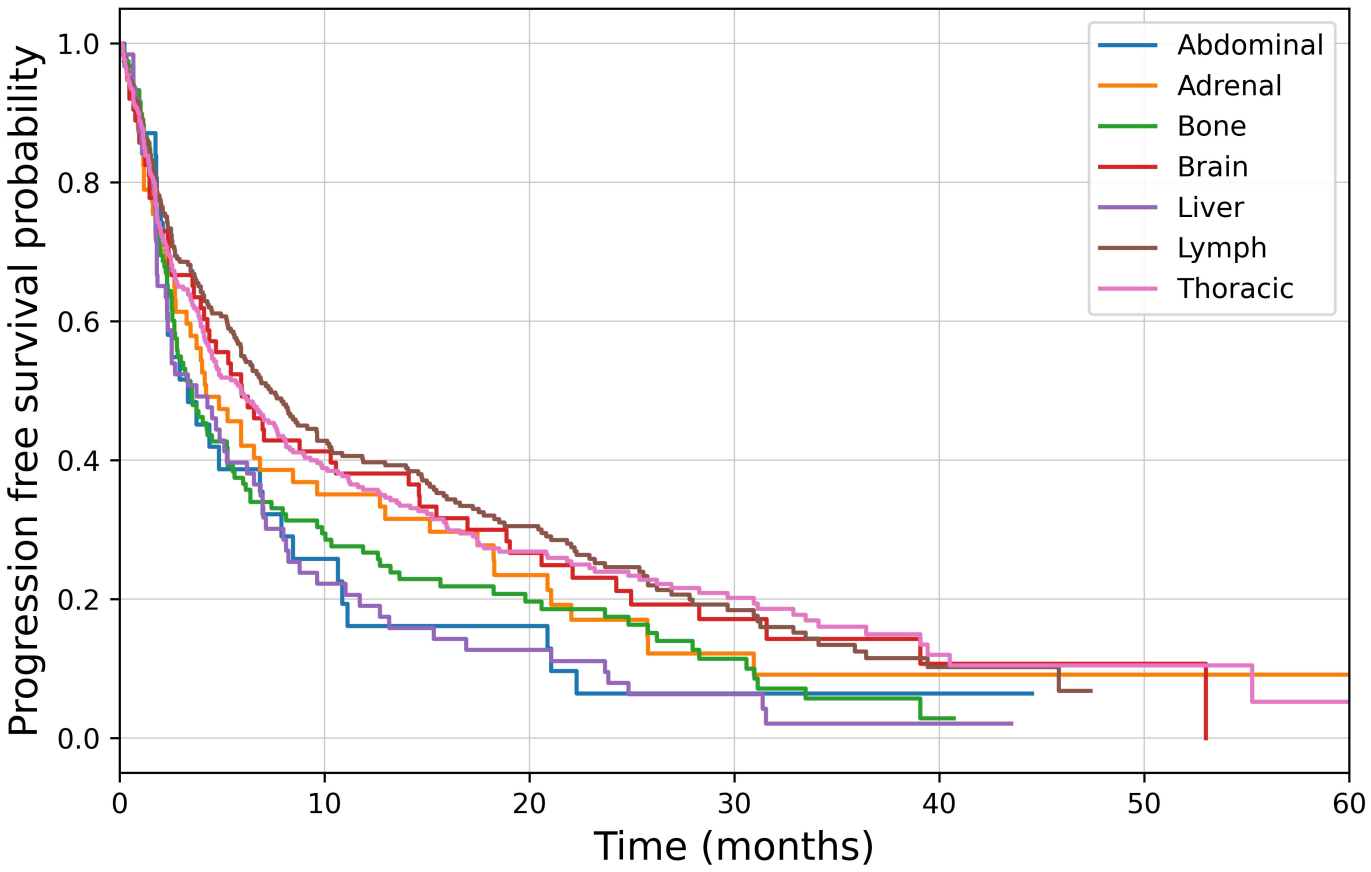
Kaplan-Meier survival analysis of organ specific progression free survival in patients with high PD-L1 expression, p<0.001.

High PD-L1 expression was present in 268 (46.8%) of patients, of which 98 (36.6%) had BoMs at baseline. In this subgroup, osPFS was significantly longer in lymph and lung lesions than the other distant sites of metastatic disease; however, no difference was observed in the osPFS in abdominal, adrenal, bone, brain and liver metastases (**Figure 2**; **Table 6**). Median PFS was 4.7 months (95%CI: 3.9–6.2) and OS was 9.1 months (95%CI: 7.3–14.8). On multivariate survival analysis, only ECOG PS ≥2 and the presence of liver metastases at baseline were identified as significant poor prognostic factors for OS (**Table 5**). Unlike in the total study cohort, BoMs were not identified as a poor prognostic factor for OS (HR: 1.24, 95%CI:0.92–1.68, p=0.16).

**Table 6:** One-year progression free survival probability by organ site of disease in patients with high PD-L1 expression.

Organ site	One year PFS (%)	Pairwise log-rank test comparison to BoMs, p-value
Abdominal	16%	0.35
Adrenal	35%	0.48
Bone	27%	
Brain	38%	0.10
Liver	19%	0.30
Lung	36%	<0.001^*^
Lymph	40%	<0.001^*^

PFS, progression free survival; BoMs, bone metastases.

### Skeletal related events

The rate of SREs amongst patients with BoMs was 44.0% (95%CI: 32.5–45). Of the patients with BoMs, 27 (11.1%) required orthopaedic surgery for an impending or actual pathologic fracture. Notably, eight of these patients requiring surgery for a BoM had PR recorded in their visceral metastases. In addition, 14 patients (5.8%) experienced spinal cord compression with four (1.5%) requiring spine surgery and 87 patients (35.8%) required RT for symptomatic BoMs.

## Discussion

ICIs are standard of care for non-oncogene driven advanced NSCLC, however, ICI efficacy in patients with BoMs is not well understood. BoMs lead to impaired quality of life through pain, fracture, and reduced mobility, thus, understanding how BoMs may be impacted by ICI is imperative to the multidisciplinary management of these patients. In this real-world study, bone, liver, and intra-abdominal lesions had a statistically shorter time to progression compared to other organ sites of disease. Significant discordance was found in the response of bone lesions versus visceral lesions. Furthermore, the presence of BoMs was an independent poor prognostic factor for OS in addition to ECOG PS ≥2, second or subsequent line of treatment, and baseline liver or other abdominal lesions. When specifically evaluating a subgroup of patients with high PD-L1 expression, it was observed that distant organ sites of disease demonstrated equivalent time to progression. In this subgroup, only ECOG PS ≥2 and liver metastases were identified as poor prognostic factors for OS on multivariate analysis. Our data validates other studies demonstrating that in advanced NSCLC, BoMs have sub-optimal disease control with ICI and patients with BoMs have poor clinical outcomes (23, 24, 28).

Differences in progression across organ sites of disease with ICI is most likely attributed to variations in the local TIME. Organ-specific TIMEs regulate tumour growth, determine metastatic progression, and likely impact the outcome of immunotherapy (27). Bone is an immunologic organ that contains an abundance of immune cells, growth factors and cytokines (41, 42). Growing evidence indicates that the bone TIME is immunosuppressive, with local immune cells unable to control cancer cell proliferation, potentially limiting the bone activity of ICIs (15, 42). The immunosuppressive TIME appears to be driven in part by the excessive bone resorption occurring with osteolytic damage, which leads to the release of immunosuppressive factors such as TGF-β and interleukin (IL)-6 production (19, 20, 22). TGF-β skews the differentiation of CD4- T cells to T helper (Th)17 polarization rather than Th1 cells, reducing the activation of effector CD8- T cell and inducing tumour-promoting inflammation (22, 42–44). TGF-β indirectly induces the expansion and activity of Tregs, enhancing their immunosuppressive activity and increasing the number seen in metastatic bone lesions (44). Tregs are highly trafficked within bone under physiologic conditions, and work to suppress the response of effector T and NK cells (34, 42). Elevated IL-6 and TGF-β further suppresses dendritic cell differentiation and NK cell proliferation (34). Altogether, this leads to exclusion of effector immune cells in the bone TIME, permitting tumour expansion and ICI resistance (20). Thus, despite the abundance of immune cells present in bone, the distinctive cytokine profile may shift the balance towards an immune-excluded TIME.

Clinical evidence supports the hypothesis that local anatomic regions of tumour growth influences the TIME composition (27). Previous studies evaluating organ specific responses to ICIs have identified the greatest response rates in lymph node metastases and poorest in liver metastases (28, 36, 45). Similar to the bone TIME, the liver appears to have distinct immune tolerance through multiple mechanisms including activation of Tregs and CD4- T cell death, affecting local and systemic tumour immunity (36). In melanoma, the presence of liver metastases has been associated with fewer tumour infiltrating lymphocytes, both at the liver lesion and at other distant metastases (46). Lymph nodes, however, are consistently responsive to ICI across studies, perhaps owing to the role of tumour-draining lymph nodes in priming antigen-specific responses to PD-1 blockade (47). Early bone response to ICI has been significantly correlated with visceral disease control (37). Using the MD Anderson criteria, Nakata et al. identified a 40% response rate of BoMs amongst 15 patients treated with nivolumab, and osteosclerotic bone response within three months of starting ICI treatment was predictive of favourable outcomes (37). Our study found time to bone progression was equivalent to liver lesions, with both anatomic sites demonstrating the shortest time to progression. This finding was also identified in a study of 761 individual lesions from patients with NSCLC that identified the worst response to nivolumab amongst bone and liver lesions (36). Further pre-clinical and clinical studies are warranted to dissect the architecture of the bone TIME and its interaction with ICI. These findings with have important therapeutic implications for guiding combination treatments and recommendations for situations of oligo-progression in future.

Studies continue to demonstrate a negative prognostic role of BoMs in NSCLC despite ICI treatment. Growing evidence supports that this is due to the immunosuppressive TIME described in bone, but other factors including large tumour burden, driver mutations, lower ECOG PS, and line of treatment may also play a role (42). In the present study, notable differences in patients with BoMs compared to those without included the presence of liver metastases, driver mutations, and line of therapy which could influence prognosis as well as response to ICI. This is a limitation of retrospective and small prospective studies evaluating this patient population. Only 1% of publications studying ICIs report on BoMs, and very few randomized trials have included outcomes pertaining to this population (42). Among patients with BoMs in the Checkmate-057 trial, death within three months of treatment initiation occurred more often in patients receiving nivolumab compared to docetaxel (1). In the Checkmate-227 trial, patients with BoMs treated with nivolumab and ipilimumab had lower median OS than patients without (13.4 months versus 18.8 months). Notably, there was no difference in median OS between the ICI treatment group and chemotherapy group for patients with BoMs (48). A retrospective study of 1959 pre-treated NSCLC patients receiving nivolumab demonstrated a significantly lower PFS and OS amongst patients with BoMs with both non-squamous and squamous histology (OS: 7.4 vs 15.3 months, p<0.001, PFS: 5 vs 10.9 months, p<0.001). These findings were irrespective of ECOG PS, presence of brain or liver disease, or prior RT to bone (24). In our study, BoMs had an independent negative impact on survival in the overall cohort in addition to ECOG, line of treatment and the presence of liver lesions.

There may be some benefit to ICI combination therapy with either chemotherapy or an EGFR-tyrosine kinase inhibitor for patients with BoMs. A retrospective study of 204 patients identified BoMs as an independent poor prognostic factor in the monotherapy group but noted no difference in clinical outcomes in the combination therapy group. While no statistical comparison was performed between treatment groups, median PFS in patients with BoMs was 4.2 months in the monotherapy group and 12.1 months in the combination group (25). Sample size constraints precluded separate analysis of patients receiving combined ICI and chemotherapy treatment in the present study. Another combination therapy with possible synergism is the use of ICI with bone modifying agents. Specifically, denosumab has been correlated with improved response to ICI and survival outcomes due to interactions with the receptor activator of nuclear factorkB pathway as well as reductions in SREs (42, 49–52). SREs are a major complication of BoMs that have an influence on quality of life, morbidity, and mortality (10). The rate of SREs in the present study was 44.0% (95%CI: 32.5–45), with 35.8% of patients receiving RT for a symptomatic bone lesion. This finding is in keeping with other studies which demonstrate an SRE rate of 30–60% in NSCLC, and may contribute to the poor response and reduced survival outcomes seen in patients with BoMs in the present study (10, 53). Furthermore, less than 10% of the study population received a bone modifying agent, precluding subgroup analysis, and this may contribute to the rate of SREs seen in the study.

PD-L1 expression is currently the only biomarker routinely available that is predictive of ICI response. Interestingly, in our study, high PD-L1 expression was associated with equivalent time to progression with ICIs amongst the distant organ sites of disease. Lung and lymph node lesions demonstrated improved osPFS, however, all other organ sites were similar. Furthermore, the presence of BoMs did not affect OS; only baseline liver metastases and poor ECOG status were poor prognostic factors. Our findings are consistent with other studies evaluating patients with high PD-L1 expression receiving first-line pembrolizumab which similarly identified liver metastases and poor ECOG status as independent predictors of poor response and survival, while other sites of disease at baseline did not affect clinical outcomes (23, 54). Tumour PD-L1 expression is unequivocally associated with favourable response to ICIs, and these findings are likely indicative of the favourable outcomes observed in this group (36, 55). This finding will have implications for local treatments such as orthopaedic stabilization, as this subgroup of patients can be expected to live longer with improved disease control. However, other factors such as line of treatment and overall tumour burden may have influenced this finding, particularly given the time of data collection when patients with high PD-L1 expression were likely to receive pembrolizumab in the first line. More studies are needed to explore the role of high PD-L1 expression on the TIME of distant sites of metastatic disease, and the corresponding responses to ICI.

Limitations of this study include the retrospective nature of the study design. We evaluated real-world data in patients not enrolled in a clinical trial, thus the frequency of follow-up imaging was variable. Many patients were pre-treated or received adjuncts to ICI including RT. Furthermore, not all patients underwent PD-L1 expression testing. Additionally, utilizing real-world data introduces heterogeneity to the study. Notable differences in patients with BoMs compared to those without included the presence of liver metastases, driver mutations, line of therapy, and number of treatment cycles, limiting the interpretation of the study findings. However, this is one of the largest studies that has evaluated the specific response of BoMs to ICI. Evaluating response of BoMs with conventional imaging strategies remains a challenge. We utilized the MDA criteria retrospectively to reduce bias, however, there remains uncertainty in categorizing bony response. The absence of a conventional chemotherapy only control arm or comparison group further limits the results. Additionally, the combination of pembrolizumab and platinum-doublet chemotherapy did not receive Health Canada approval until 2019 therefore a relatively small proportion of patients in this study were offered combination treatment. This precluded subgroup analysis of bone response to combination treatment. Additional studies in larger cohorts are needed to further refine our understanding of the bone TIME and responsiveness to ICI.

Our study demonstrated inferior clinical responses in metastatic bone lesions relative to other sites of disease. BoMs in our cohort did not respond favourably to ICI and were associated with inferior clinical outcomes. The effect of BoMs on disease progression and OS with ICI is an understudied area in the immune-oncology literature. BoMs are a considerable source of pain, morbidity, and mortality in patients with advanced cancer. As the indications for immunotherapies continue to increase, it will be important to further understand the bone TIME of metastatic lesions and explore how the local bone immune biology may be targeted to bolster responses to ICI.

## Data availability statement

The datasets presented in this article are not readily available because while possible, institutional and research ethics board approval would be required for data sharing. Requests to access the datasets should be directed to annalise.abbott@ucalgary.ca.

## Ethics statement

The studies involving humans were approved by Health Research Ethics Board of Alberta Cancer Committee-19-0380. The studies were conducted in accordance with the local legislation and institutional requirements. Written informed consent for participation was not required from the participants or the participants' legal guardians/next of kin in accordance with the national legislation and institutional requirements.

## Author contributions

AA: Conceptualization, Data curation, Formal analysis, Funding acquisition, Investigation, Methodology, Writing – original draft, Writing – review & editing. DM: Conceptualization, Data curation, Methodology, Writing – review & editing, Formal analysis. GE: Formal analysis, Methodology, Supervision, Visualization, Writing – review & editing. IS: Conceptualization, Data curation, Investigation, Methodology, Writing – review & editing. AM: Conceptualization, Data curation, Investigation, Methodology, Writing – review & editing. SP: Conceptualization, Funding acquisition, Investigation, Methodology, Supervision, Writing – review & editing. DM: Conceptualization, Investigation, Methodology, Supervision, Writing – review & editing. WC: Conceptualization, Formal analysis, Methodology, Supervision, Writing – review & editing. MM: Conceptualization, Data curation, Funding acquisition, Investigation, Methodology, Supervision, Writing – review & editing.

## References

[1] Borghaei H Paz-Ares L Horn L Spigel DR Steins M Ready NE. Nivolumab versus docetaxel in advanced nonsquamous non-small-cell lung cancer. N Engl J Med. (2015) 373:1627–39. doi: 10.1056/NEJMoa1507643 PMC5705936 26412456

[2] Brahmer J Reckamp KL Bass P Crinó L Eberhardt WEE Poddubskaya E. Nivolumab versus docetaxel in advanced squamous cell non-small-cell lung cancer. N Engl J Med. (2015) 373:123–35. doi: 10.1056/NEJMoa1504627 PMC4681400 26028407

[3] Gandhi L Rodriguez-Abreu D Gadgell S Esteban E Felip E De Angelis F. Pembrolizumab plus chemotherapy in metastatic non-small-cell lung cancer. N Engl J Med. (2018) 378:2078–92. doi: 10.1056/NEJMoa1801005 29658856

[4] Garon EB Hellmann MD Rizvi NA Carcereny E Leighl NB Ahn M. Five-year overall survival for patients with advanced non-small-cell lung cancer treated with pembrolizumab: Results from the Phase I KEYNOTE-001 study. J Clin Oncol. (2019) 37:2518–27. doi: 10.1200/JCO.19.00934 PMC6768611 31154919

[5] Herbst RS Bas P Kim D Felip E Pérez-Gracia J Han J. Pembrolizumab versus docetaxel for previously treated, PD-L1-positive advanced non-small-cell lung cancer (KEYNOTE-010): a randomised controlled trial. Lancet. (2016) 387:1540–50. doi: 10.1016/S0140-6736(15)01281-7 26712084

[6] Mok TSK Wu Y Kudaba I Kowalski DM Chul Cho B Turna HZ. Pembrolizumab versus chemotherapy for previously untreated, PD-L1-expressing locally advanced or metastatic non-small-cell lung cancer (KEYNOTE-042): a randomised, open-label, controlled, phase 3 trial. Lancet. (2019) 393:1819–30. doi: 10.1016/S0140-6736(18)32409-7 30955977

[7] Reck M Rodriguez-Abreu D Robinson AG Hui R Csószi T Fülöp A. Updated analysis of KEYNOTE-024: pembrolizumab versus platinum-based chemotherapy for advanced non–small-cell lung cancer with PD-L1 tumor proportion score of 50% or greater. J Clin Oncol. (2019) 2019:37: 537–546. doi: 10.1200/JCO.18.00149 30620668

[8] Langer JL. Emerging immunotherapies in the treatment of non-small cell lung cancer (NSCLC): The role of immune checkpoint inhibitors. Am J Clin Oncol. (2015) 38:422–30. doi: 10.1097/COC.0000000000000059 24685885

[9] Pardoll DM. The blockade of immune checkpoints in cancer immunotherapy. Nat Rev Cancer. (2012) 12(4):252–64. doi: 10.1038/nrc3239 PMC4856023 22437870

[10] Decroisette C Monnet I Berard H Quere G Le Caer H Bota S. Epidemiology and treatment costs of bone metastases from lung cancer: A french prospective, observational, multicenter study (GFPC 0601). J Thorac Oncol. (2011) 6:576–82. doi: 10.1097/JTO.0b013e318206a1e3 21270669

[11] Mayorca-Guiliani AE Madsen CD Cox TR Horton ER Venning FA Erler JT. ISDoT: in *situ* decellularization of tissues for high-resolution imaging and proteomic analysis of native extracellular matrix. Nat Med. (2017) 23:890–8. doi: 10.1038/nm.4352 28604702

[12] Peng DH Rodriguez BL Diao L Chen L Wang J Byers LA. Collagen promotes anti-PD-1/PD-L1 resistance in cancer through LAIR1-dependent CD8 T cell exhaustion. Nat Commun. (2020) 11:4520. doi: 10.1038/s41467-020-18298-8 32908154 PMC7481212

[13] Roato I. Bone metastases: When and how lung cancer interacts with bone. World J Clin Oncol. (2014) 5:149. doi: 10.5306/wjco.v5.i2.149 24829862 PMC4014787

[14] Roodman GD. Mechanisms of bone metastasis. N Engl J Med. (2004) 350:1655–64. doi: 10.1056/NEJMra030831 15084698

[15] Passaro A Attili I Morganti S. Clinical features affecting survival in metastatic NSCLC treated with immunotherapy: A critical review of published data. Cancer Treat Rev. (2020) 89:1–10. doi: 10.1016/j.ctrv.2020.102085 32771858

[16] Yang K Li J Bai C. Efficacy of immune checkpoint inhibitors in non-small-cell lung cancer patients with different metastatic sites: A systematic review and meta-analysis. Front Oncol. (2020) 10:1098. doi: 10.3389/fonc.2020.01098 32733805 PMC7363957

[17] Botticelli A Cirillo A Scagnoli S Del Signore E Gianoncelli L Spitaleri G. The agnostic role of site of metastasis in predicting outcomes in cancer patients treated with immunotherapy. Vaccines. (2020) 8(203):1–21. doi: 10.3390/vaccines8020203 PMC7349154 32353934

[18] Bussard KM Venzon DJ Mastro AM. Osteoblasts are a major source of inflammatory cytokines in the tumor microenvironment of bone metastatic breast cancer. J Cell Biochem. (2010) 111:1138–48. doi: 10.1002/jcb.22799 PMC3654838 20683902

[19] Fournier PGJ Chirgwin JM Guise TA. New insights into the role of T cells in the vicious cycle of bone metastases. Curr Opin Rheumatol. (2006) 18:396–404. doi: 10.1097/01.bor.0000231909.35043.da 16763461

[20] Hensel JA Khattar V Ashton R Lee C Siegal GP Ponnazhagan S. Location of tumor affects local and distant immune cell type and number. Immun Inflamm. Dis. (2017) 5:85–94. doi: 10.1002/iid3.144 28250928 PMC5322166

[21] Ihle CL Owens P. Integrating the immune microenvironment of prostate cancer induced bone disease. Mol Carcinogen. (2020) 59:822–9. doi: 10.1002/mc.23192 32233011

[22] Jiao S Subudhi SK Aparicio A Ge Z Guan B Miura Y. Differences in tumor microenvironment dictate T helper lineage polarization and response to immune checkpoint therapy. Cell. (2019) 179:1170–90. doi: 10.1016/j.cell.2019.10.029 31730856

[23] Kawachi H Tamiya M Tamiya A Ishii S Hirano K Matsumoto H. Association between metastatic sites and first-line pembrolizumab treatment outcome for advanced non–small cell lung cancer with high PD-L1 expression: a retrospective multicenter cohort study. Invest New Drugs. (2020) 38:211–8. doi: 10.1007/s10637-019-00882-5 31784866

[24] Landi L D’Inca F Gelibter A Chiari R Grossi F Delmonte A. Bone metastases and immunotherapy in patients with advanced non-small-cell lung cancer. J Immunother Cancer. (2019) 7:316–25. doi: 10.1186/s40425-019-0793-8 PMC6868703 31752994

[25] Li J Zhu H Sun L Xu W Wang X. Prognostic value of site-specific metastases in lung cancer: A population based study. J Cancer. (2019) 10:3079–86. doi: 10.7150/jca.30463 PMC6603375 31289577

[26] Li X Wang L Chen S Zhou F Zhao J Zhao W. Adverse impact of bone metastases on clinical outcomes of patients with advanced non-small-cell lung cancer treated with immune checkpoint inhibitors. Thorac Cancer. (2020) 11:2812–9. doi: 10.1111/1759-7714.13597 PMC7529562 32779372

[27] Oliver AJ Lau PKH Unsworth AS Loi S Darcy PK Kershaw MH. Tissue-dependent tumor microenvironments and their impact on immunotherapy responses. Front Immunol. (2018) 9:1–8. doi: 10.3389/fimmu.2018.00070 29445373 PMC5797771

[28] Schmid S Diem S Li Q Krapf M Flatz L Leschka S. Organ-specific response to nivolumab in patients with non-small cell lung cancer. Cancer Immunol Immun. (2018) 67:1825–32. doi: 10.1007/s00262-018-2239-4 PMC11028265 30171269

[29] Horton BL Fessenden TB Spranger S. Tissue site and the cancer immunity cycle. Trends Cancer. (2019) 5:593–603. doi: 10.1016/j.trecan.2019.07.006 31706507 PMC7521621

[30] Oliver AJ Davey AS Keam SP Mardiana S Chan JD von Scheidt B. Tissue-specific tumor microenvironments influence responses to therapies. Clin Transl Immunol. (2019) 8:e1094. doi: 10.1002/cti2.1094 PMC6869967 31768254

[31] Salmon H Franciszkiewicz K Damotte D Dieu-Nosjean MC Validire P Trautmann A. Matrix architecture defines the preferential localization and migration of T cells into the stroma of human lung tumors. J Clin Invest. (2012) 122:899–910. doi: 10.1172/JCI45817 22293174 PMC3287213

[32] Salmon H Remark R Gnjatic S Merad M. Host tissue determinants of tumor immunity. Nat Rev Cancer. (2019) 19:215–27. doi: 10.1038/s41568-019-0125-9 PMC7787168 30867580

[33] Roato I Ferracini R. Cancer stem cells, bone and tumor microenvironment: key players in bone metastases. Cancers. (2018) 10:56. doi: 10.3390/cancers10020056 29461491 PMC5836088

[34] Koh BI Kang Y. The pro-metastatic role of bone marrow-derived cells: a focus on MSCs and regulatory T cells. EMBO Rep. (2012) 13:412–22. doi: 10.1038/embor.2012.41 PMC3343352 22473297

[35] Liu C Wang M Changli X Li B Chen J Chen J. Immune checkpoint inhibitor therapy for bone metastases: Specific microenvironment and current situation. J Immunol Res. (2021), 1–18. doi: 10.1155/2021/8970173 PMC8645368 34877360

[36] Osorio JC Arbour KC Le DT Durham JN Plodkowski AJ Halpenny DF. Lesion-level response dynamics to programmed cell death protein (PD-1) blockade. J Clin Oncol. (2019) 37:3546–55. doi: 10.1200/JCO.19.00709 PMC7194449 31675272

[37] Nakata E Sugihara S Sugawara Y Kozuki T Harada D Nogami N. Early response of bone metastases can predict tumor response in patients with non-small-cell lung cancer with bone metastases in the treatment with nivolumab. Oncol Lett. (2020) 20:2977–86. doi: 10.3892/ol.2020.11856 PMC7401004 32782615

[38] Meyers DE Stukalin I Vallerand IA Lewinson RT Suo A Dean M. The lung immune prognostic index discriminates survival outcomes in patients with solid tumors treated with immune checkpoint inhibitors. Cancers. (2019) 11:1713. doi: 10.3390/cancers11111713 31684111 PMC6896022

[39] Eisenhauer EA Therasse P Bogaerts J Schwartz LH Sargent D Ford R. New response evaluation criteria in solid tumours: Revised RECIST guideline (version 1.1). Eur J Cancer. (2009) 45:228–47. doi: 10.1016/j.ejca.2008.10.026 19097774

[40] Hamaoka T Costelloe CM Madewell JE Liu P Berry DA Islam R. Tumour response interpretation with new tumour response criteria vs the World Health Organization criteria in patients with bone-only metastatic breast cancer. Brit J Cancer. (2010) 102:651–7. doi: 10.1038/sj.bjc.6605546 PMC2837571 20104228

[41] Zhao E Xu H Wang L Kryczek I Wu K Hu Y. Bone marrow and the control of immunity. Cell Mol Immunol. (2012) 9:11–9. doi: 10.1038/cmi.2011.47 PMC3251706 22020068

[42] Del Conte A De Carlo E Bertoli E Stanzione B Revelant A Bertola M. Bone metastasis and immune checkpoint inhibitors in non-small-cell lung cancer (NSCLC): Microenvironment and possible clinical implications. Int J Mol Sci. (2022) 23:6832. doi: 10.3390/ijms23126832 35743275 PMC9224636

[43] Mariathasan S Turley SJ Nickles D Castiglioni A Yuen K Wang Y. TGFβ attenuates tumour response to PD-L1 blockade by contributing to exclusion of T cells. Nature. (2018) 554:544–8. doi: 10.1038/nature25501 PMC6028240 29443960

[44] Wrzesink Wrzesinski SH Wan YY Flavell RA. Transforming growth factor-β and the immune response: implications for anticancer therapy. Clin Cancer Res. (2007) 13:5262–70. doi: 10.1158/1078-0432.CCR-07-1157 17875754

[45] Nishino M Ramaiya NH Chambers ES Adeni AE Hatabu H Jänne PA. Immune-related response assessment during PD-1 inhibitor therapy in advanced non-small-cell lung cancer patients. J Immunother Cancer. (2016) 4:84. doi: 10.1186/s40425-016-0193-2 28018599 PMC5168591

[46] Tumeh P Hellmann M Hamid O Tsai KK Loo KL Gubens MA. Liver metastasis and treatment outcome with anti-PD-1 monoclonal antibody in patients with melanoma and NSCLC. Cancer Immunol Res. (2017) 5:417–24. doi: 10.1158/2326-6066.CIR-16-0325 PMC5749922 28411193

[47] Stachura J Wachowska M Kilarski WW Güç E Golab J Muchowicz A. The dual role of tumor lymphatic vessels in dissemination of metastases and immune response development. Oncoimmunology. (2016) 5:1–10. doi: 10.1080/2162402X.2016.1182278 PMC5006909 27622039

[48] Hellmann MD Paz-Ares L Bernabe Caro R Zurawski B Kim SW Carcereny Costa E. Nivolumab plus ipilimumab in advanced non-small-cell lung cancer. N Engl J Med. (2019) 381:2020–31. doi: 10.1056/NEJMoa1910231 31562796

[49] Asano Y Yamamoto N Demura S Hayashi K Takeuchi A Kato S. The therapeutic effect and clinical outcome of immune checkpoint inhibitors on bone metastasis in advanced non-small-cell lung cancer. Front Oncol. (2022) 12. doi: 10.3389/fonc.2022.871675 PMC9010859 35433422

[50] Asano Y Yamamoto N Demura S Hayashi K Takeuchi A Kato S. Novel predictors of immune checkpoint inhibitors response and prognosis in advanced non-small-cell lung cancer with bone metastasis. Cancer Med. (2023) 12:12425–37. doi: 10.1002/cam4.5952 PMC10278505 37076988

[51] Li H Lei S Li J Xing P Hao X Xu F. Efficacy and safety of concomitant immunotherapy and denosumab in patients with advanced non-small-cell lung cancer carrying bone metastases: A retrospective chart review. Front Immunol. (2022) 13. doi: 10.3389/fimmu.2022.908436 PMC9464943 36105807

[52] Bongiovanni A Foca F Menis J Luigia Stucci S Artioli F Guadalupi V. Immune checkpoint inhibitors with or without bone-targeted therapy in NSCLC patients with bone metastases and prognostic significance of neutrophil-to-lymphocyte ratio. Front Immunol. (2021) 12. doi: 10.3389/fimmu.2021.697298 PMC8631508 34858389

[53] Kuchuk M Addison CL Clemons M Kuchuk I Wheatley-Price P. Incidence and consequences of bone metastases in lung cancer patients. J Bone Oncol. (2013) 2:22–9. doi: 10.1016/j.jbo.2012.12.004 PMC4723355 26909268

[54] Takeyatsu Y Yoshida T Shibaki R Matsumoto Y Goto Y Kanda S. Differential efficacy of pembrolizumab according to metastatic sites in patients with PD-L1 strongly positive (TPS ≥50%) NSCLC. Clin Lung Cancer. (2020) 12(2):127–133. doi: 10.1016/j.cllc.2020.10.002 33183972

[55] Zou Y Hu X Zheng S Yang A Li X Tang H. Discordacne of immunotherapy response predictive biomarkers between primary lesions and paired metastases in tumours: A multidimensional analysis. Ebiomedicine. (2020) 63:1–15. doi: 10.1016/j.ebiom.2020.103137 PMC7736926 33310681

